# Supplementary material for: Effect of Selected Antidepressants on Placental Homeostasis of Serotonin: Maternal and Fetal Perspectives
Source: Pharmaceutics. 2021 Aug 20;13(8):1306. doi: 10.3390/pharmaceutics13081306 (PMC8397948; doi:10.3390/pharmaceutics13081306)
Supplement: Supplementary file 1 [file pharmaceutics-13-01306-s001.zip › pharmaceutics-1318913-supplementary.pdf]

pharmaceutics

Supplementary Materials

Effect of Selected Antidepressants on Placental Homeostasis of Serotonin: Maternal and Fetal Perspectives

Hana Horackova, Rona Karahoda, Lukas Cerveny, Veronika Vachalova, Ronja Ebner, Cilia Abad and Frantisek Staud *

Table S1. Purity and orientation of microvillous (MVM) and basal (BM) membranes isolated from human term placenta. Data shown are means ± SD, n ≥ 4; the enrichment factors (EF) were calculated relative to starting placental homogenate.

Parameter	MVM	BM
Orientation (% right side out)	96.12 ± 7.35	81.03 ± 3.69
Alkaline phosphatase (fold enrichment)	15.09 ± 6.4	4.67 ± 1.33
Dihydroalprenolol binding (fold enrichment)	9.73 ± 5.8	29.15 ± 12.37
